# Autoinflammation with infantile enterocolitis induced by a heterozygous variant (c.1357C > T) in the NLRC4 gene: a case report

**DOI:** 10.3389/fped.2026.1822554

**Published:** 2026-05-28

**Authors:** Xing Wang, Yongmei Xiao, Ting Ge, Ting Zhang, Xiaolu Li

**Affiliations:** 1 Department of Gastroenterology, Hepatology and Nutrition, Shanghai Children’s Hospital, Shanghai Jiao Tong University, Shanghai, China; 2 Institue of Pediatric Infection, Immunity and Critical Care Medicine, Shanghai Children’s Hospital, Shanghai Jiao Tong University School of Medicine, Shanghai, China

**Keywords:** enterocolitis autoinflammatory diseases, gain-of-function, infant, necrotizing enterocolitis, NLRC4 protein human mutation

## Abstract

**Background:**

Necrotizing enterocolitis (NEC) is s a life-threatening inflammatory intestinal disorder primarily affecting preterm infants, though rare in full-term neonates. NLR-family CARD domain-containing protein 4 (*NLRC4*), a cytosolic inflammasome component driving IL-1β-mediated inflammation, is linked to autoinflammatory syndromes via germline gain-of-function mutations, though its role in term infant NEC remains underexplored.

**Case presentation:**

A full-term male infant developed NEC shortly after birth, requiring emergency surgical interventions including ileostomy and intestinal resection. Intraoperative findings revealed multifocal necrosis in the small intestine (80 cm total length) and colon. Genetic testing identified a heterozygous *NLRC4* variant (c*.1357C* *>* T, p. Arg 453*), inherited from an asymptomatic father. Sanger sequencing confirmed the mutation's *de novo* origin. Pathological analysis demonstrated transmural inflammation without evidence of Hirschsprung disease.

**Conclusions:**

This report identifies a novel truncating variant in the *NLRC4* gene associated with severe autoinflammatory enterocolitis presenting as NEC in a term neonate. Early *NLRC4* screening may guide targeted therapies and improve outcomes in severe intestinal inflammation.

## Background

Necrotizing enterocolitis (NEC) is a severe intestinal disease of neonates, predominantly affecting preterm and very low birth weight infants. Its pathogenesis, which involves gut immaturity, dysbiosis, enteral feeding, and a dysregulated immune response, is not fully defined. The disease is marked by intestinal inflammation and necrosis, usually presenting after the initiation of enteral feeds and carrying risks of perforation, sepsis, and death (1). Epidemiologically, NEC is a leading cause of morbidity and mortality in neonatal intensive care units. The incidence among VLBW infants is approximately 7%. Mortality is significant, reported at 23.5% for all neonates with confirmed NEC, increasing to 34.5% in those requiring surgery, and exceeding 40%–50% for extremely low birth weight (<1000 g) infants, particularly those undergoing surgical intervention. Data from China align with this trend, showing an incidence of 5.5% in infants weighing <1500 g and 7.0% in those <1000 g (2–4).

NLRC4, a cytosolic innate immune sensor primarily expressed in macrophages and intestinal epithelial cells, functions by activating the inflammasome to drive the maturation and secretion of interleukin-1β (IL-1β) (5). The NLRC4gene is located on chromosome 2p21-p22, consists of nine exons, and is transcriptionally regulated by p53 and pro-inflammatory signals such as TNF-α (6). NLRC4 inflammasomopathies comprise a spectrum of auto inflammatory diseases driven by gain-of-function (GOF) mutations. The molecular mechanisms vary by mutation location: mutations in the nucleotide-binding domain (NBD) promote inflammasome aggregation and hyperresponsiveness, whereas mutations or loss of the leucine-rich repeat (LRR) regulatory domain result in sustained inflammasome activation (7). The associated clinical phenotypes are broad, ranging from cold urticaria and neonatal-onset multisystem inflammatory disease (NOMID) to the frequently fatal autoinflammation with infantile enterocolitis (AIFEC). Since 2014, a total of 34 patients with NLRC4 GOF mutation-associated diseases have been reported (8). The NLRC4 inflammasome, a component of the innate immune system, mediates the cleavage and release of the pro-inflammatory cytokines IL-1β and IL-18, thereby driving inflammatory responses. GOF mutations in NLRC4 are clinically associated with early-onset recurrent fever, recurrent macrophage activation syndrome, and enterocolitis (7). These mutations have been linked to early-onset recurrent fever, recurrent macrophage activation syndrome(MAS), enterocolitis, and cancer susceptibility (9). The NLRC4 (A160T) variant can cause recessive immune dysregulation/autoinflammation or act as a heterozygous risk factor for ulcerative colitis, often affecting epithelial cells and colon tissue (10). In this study, we report a novel truncating NLRC4 variant, p.Arg453*, located in the LRR domain, in a Chinese term infant with severe enterocolitis. This finding expands the genotypic and phenotypic spectrum of NLRC4-associated disorders, highlighting its role in severe infantile enterocolitis even in term neonates.

## Case presentation

A full-term male neonate (birth weight 2950 g) was delivered via spontaneous vaginal delivery to a primigravida (G1P1). Intrapartum monitoring was unremarkable. The infant was formula-fed after birth. On the third postnatal day, the infant was admitted to the neonatal intensive care unit with a provisional diagnosis of neonatal hyperbilirubinemia. During hospitalization, the patient passed dark red bloody stools accompanied by abdominal distension, hypoactive bowel sounds (4/min), and decreased responsiveness. Investigations revealed elevated inflammatory markers and stress-induced hyperglycemia. Abdominal radiography demonstrated irregular luminal contours, diffuse bowel wall thickening, and hyperechoic foci within the intestinal wall suggestive of pneumatosis intestinalis.

The infant was immediately managed with nil per os (NPO), intravenous fluid resuscitation, and nasogastric decompression, followed by emergent exploratory laparotomy under general anesthesia. Procedures included ileostomy, ileal resection, adhesiolysis, and percutaneous abdominal drainage. Intraoperative examination revealed a markedly shortened small intestine with a total length of 80 cm. A segment of the distal ileum, spanning 65 cm in length and located beginning at 15 cm proximal to the ileocecal valve, exhibited diffuse patchy mucosal necrosis without transmural involvement. Within this segment, a full-thickness necrotic lesion measuring 2.5 × 1 cm with perforation was identified 1 cm proximal to the ileocecal junction. The remaining jejunum and proximal ileum appeared grossly normal. The patient was successfully discharged at 5 months of age with scheduled outpatient follow-up. He is currently 5 years old and has undergone regular follow-up since discharge. During the follow-up period, he has not experienced diarrhea, his weight gain has remained within the normal range, the abdominal enterostomy has been closed, and there have been no recurrent fevers or further episodes of enterocolitis. Pathological analysis confirmed transmural inflammation. Although pathological analysis did not demonstrate evidence of Hirschsprung's disease (HD), we cannot definitively rule out this condition based on current findings.

**Figure 1:**
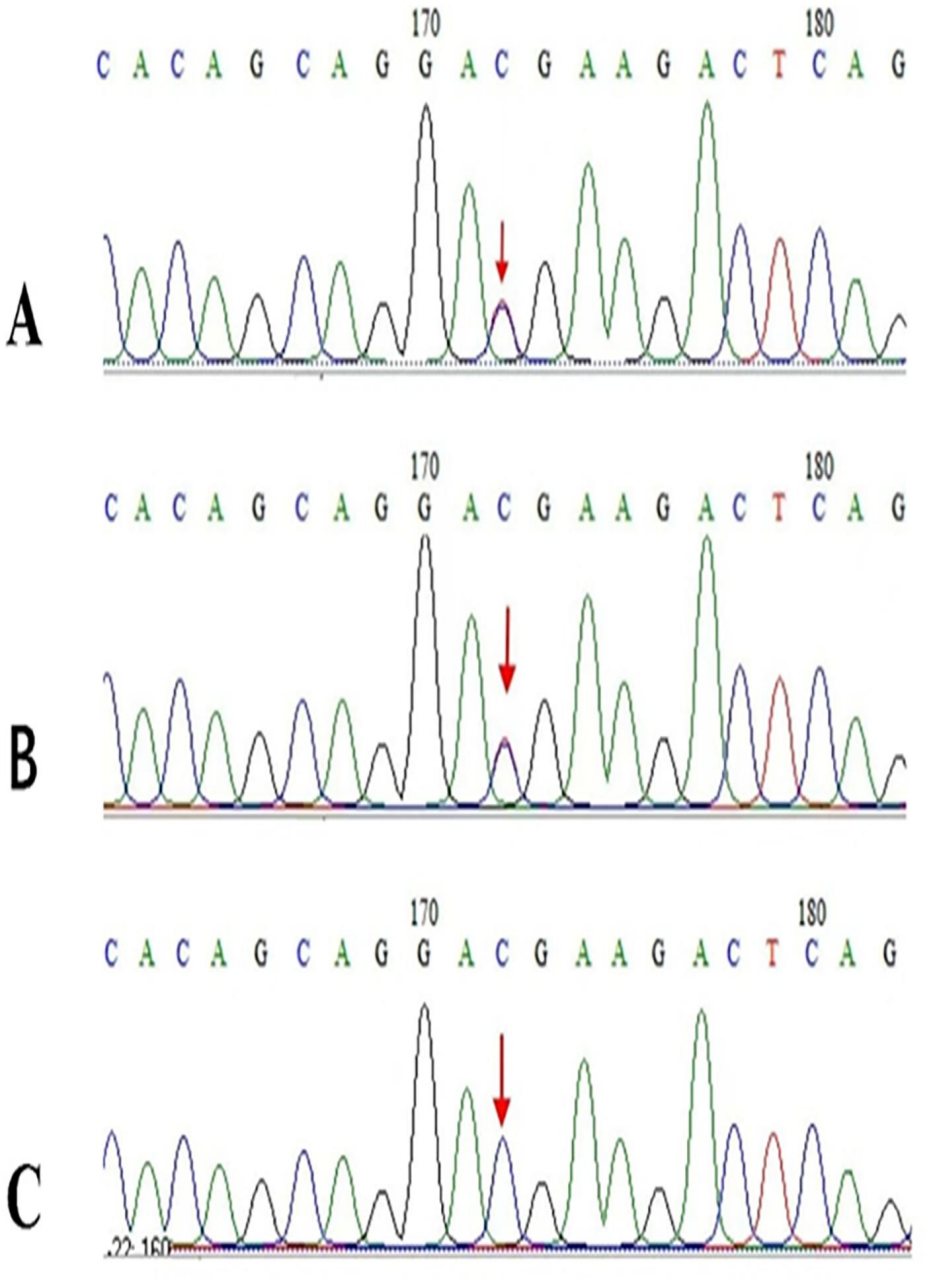
Sanger sequencing chromatograms of the proband and parents. **A**: Proband: Heterozygous NLRC4 variant (c.1357C > T, p.Arg453*) (arrow) **B**: Father: Heterozygous NLRC4 variant (c.1357C > T, p.Arg453*) (arrow) **C**: Mother: Wild-type sequence at the indicated locus (arrow).

Genetic testing was offered due to the atypical presentation of NEC in a full-term infant with severe and early-onset disease. Approval for this genetic testing was obtained from the ethics committee of Shanghai Children's Hospital(2026R035-E01). Genomic DNA was extracted from peripheral blood leukocytes using the QIAamp DNA Blood Mini Kit (Qiagen, Germany). Whole-exome sequencing was performed on the Illumina NovaSeq 6000 platform with 150 bp paired-end reads. Raw data were processed through a standard bioinformatics pipeline, including alignment to the GRCh37/hg19 reference genome using BWA, variant calling with GATK, and annotation with ANNOVAR. Variants were filtered based on population frequency (<1% in gnomAD), predicted pathogenicity, and clinical relevance. Segregation analysis was performed on available family members to establish inheritance patterns Genomic analysis identified a heterozygous pathogenic variant in the *NLRC4* gene (c.1357C > T) through whole exome sequencing of the proband's whole blood DNA (Figure 1). Segregation analysis established paternal inheritance of this germline mutation. The variant is novel to the Human Gene Mutation Database (HGMD) and exhibits an ultra-rare minor allele frequency (<0.01%) in the gnom AD population database, with maximal representation in the American cohort (0.0145%). No homozygous carriers have been documented in global genomic databases. In accordance with the American College of Medical Genetics and Genomics (ACMG) variant interpretation guidelines. This variant was classified as potentially clinically significant given its correlation with the patient's disease phenotype.

## Discussion

Emerging evidence underscores a growing recognition of the pivotal role played by genetic predisposition in the pathogenesis of NEC. Reported gene mutations associated with NEC are summarized in the tables (Supplementary Tables 1, 2). NEC causes inflammatory bowel necrosis, placing infants at risk for neurodevelopmental impairment, short bowel syndrome with or without intestinal failure (IF), intestinal strictures, and cholestatic liver disease driven by chronic parenteral nutrition (PN) use and inflammation (11). IN this case, a rectal biopsy was not performed because the patient's clinical presentation (severe enterocolitis with perforation requiring emergent surgery) and the macroscopic findings during laparotomy were highly suggestive of NEC in the context of an *NLRC4* inflammasomopathy, rather than classic HD. Furthermore, the pathological examination confirmed transmural inflammation in the resected ileum, a feature not typical of uncomplicated HD (12). HD cannot be definitively excluded without a rectal biopsy, and in future cases with overlapping features, a rectal biopsy or calretinin immunohistochemistry should be considered if clinical suspicion for HD persists. Contemporary evidence synthesis demonstrates that genetic predisposition constitutes a fundamental etiological component in neonatal NEC, supported by twin studies, genome-wide association studies (GWAS), and candidate gene investigations, particularly in term and late-preterm infants. Mounting evidence supports melatonin's therapeutic effect on NEC, potentially through AMPK/SIRT1 signaling-mediated regulation of the Th17/Treg cell balance (13). Chen Zhenhui's team (14) demonstrated that *Bacteroides fragilis* ameliorates NEC via bile salt hydrolase (BSH) activity, suppressing the FXR-NLRP3 pathway and correcting gut dysbiosis and bile acid disturbances. Mechanistically, taurochenodeoxycholic acid exacerbates NEC, while taurodoxycholic acid shows efficacy. Dr. Saravanan Subramanian's team (15) discovered that tilivalline disrupts microbiota-activated STAT1 signaling controlling *NLRC5* expression in intestinal epithelial cells (IECs) through a PPAR-*γ*-mediated mechanism, hindering resistance to self-NK1.1 + cell cytotoxicity and promoting NEC upon viral challenge/colonization, highlighting microbiota's role in shaping disease tolerance.

**Figure 2:**
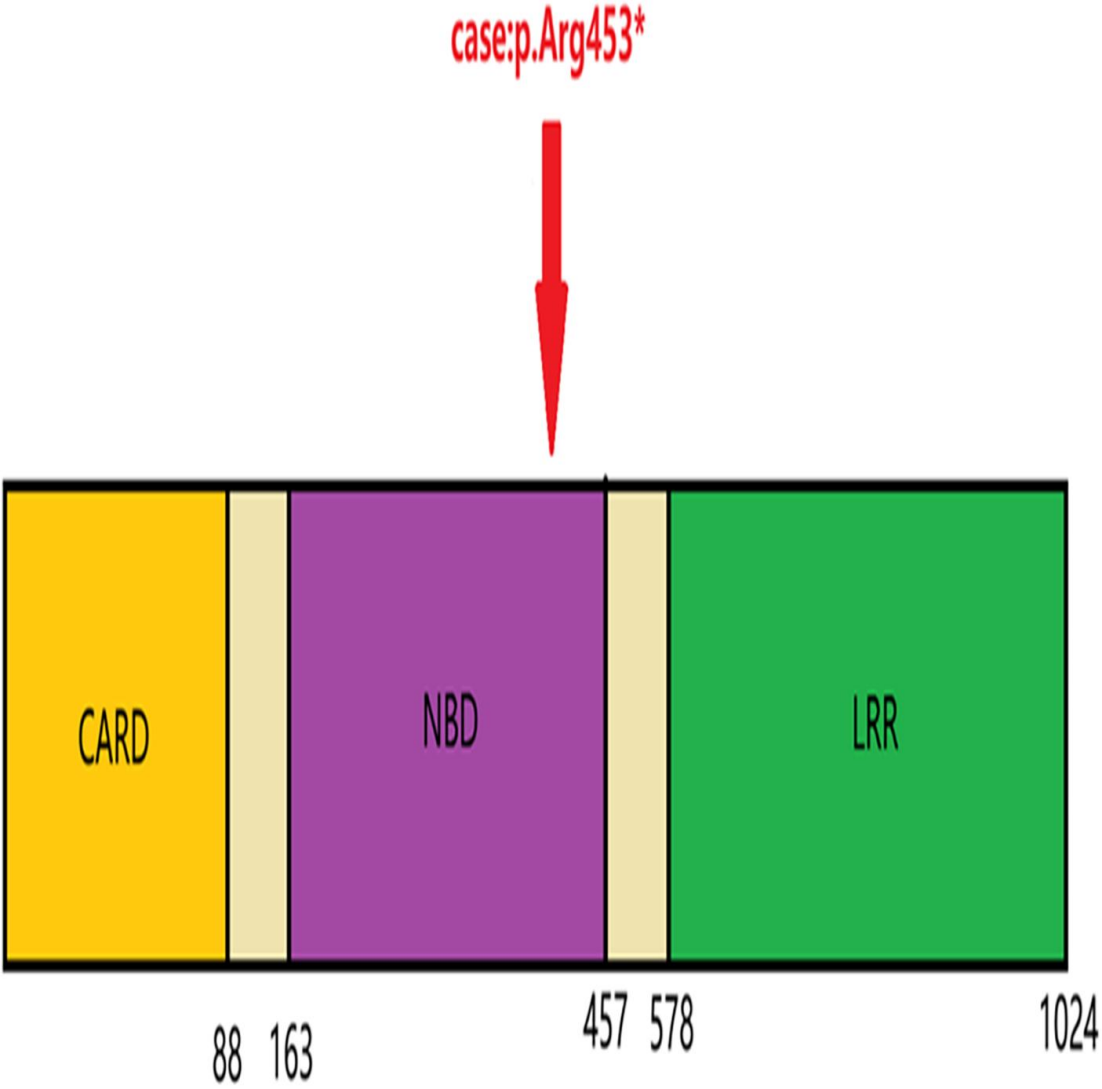
Schematic representation of the NLRC4 protein domains. CARD, N-terminal caspase activation and recruitment domain; NBD, central nucleotide-binding domain; LRR, C-terminal leucine-rich repeat domain; Red arrow, location of the novel mutation c.1357C > T (p.Arg453*).

The *NLRC4* gene (chromosome 2p21-p22, 9 exons) encodes a 1024-amino acid protein (111 kDa) characterized by three key domains: an N-terminal caspase activation and recruitment domain (CARD), a central nucleotide-binding domain (NBD), and a C-terminal LRR domain, which acts as a regulatory element (Figure 2). These pathogenic mechanisms underpin a spectrum of disorders categorized into four groups: AIFEC, MAS, neonatal-onset multisystem inflammatory disease (NOMID), and familial cold autoinflammatory syndrome 4 (FCAS4), with AIFEC characterized by chronic inflammation and episodes of extreme acuity (8) *6*) (16). MAS-like manifestations are common across mutations in either domain, while NBD mutations have also been linked specifically to arthritis or FCAS4 (7). Management typically targets downstream inflammatory mediators like IL-1β due to the current lack of specific *NLRC4* inhibitors (8). Although nonsense mutations often trigger NMD, certain truncating mutations can escape degradation and produce stable, hyperactive proteins—a phenomenon documented in related genes such as *NLRP3* (17). Several factors support a GOF mechanism in this case: the variant occurs in the autoinhibitory LRR domain of *NLRC4*, likely disrupting regulatory control and promoting spontaneous activation (18); and while functional validation remains necessary, the severity of the phenotype suggests a GOF effect beyond haploinsufficiency. GOF mutations in *NLRC4* can cause constitutive inflammasome activation and excessive IL-1β/IL-18 secretion, even in the absence of pathogens, thereby amplifying inflammatory tissue injury and mimicking aspects of TLR-mediated hyperinflammation. Conversely, impaired *NLRC4* function (as may occur in LOF or partial LOF contexts) could diminish bacterial clearance, facilitating Gram-negative bacterial overgrowth and subsequent TLR activation. This cross-talk between *NLRC4* and *TLR* pathways—particularly in response to flagellin and other conserved microbial motifs (19, 20).

## Conclusions

In the present case, whole-exome sequencing identified a heterozygous nonsense/truncating variant (c.1357C > T, p.Arg453*) in the proband. Segregation analysis confirmed this variant was paternally inherited. This variant is novel (absent from HGMD) and exhibits extreme rarity in population databases (gnomAD overall AF: 0.0032% [8/251,448], highest in American subpopulation: 0.0145% [3/34,584], no homozygotes). Classified as potentially clinically significant per ACMG guidelines, its identification aligns with an inflammasomopathy phenotype. Critically, the p.Arg453* mutation introduces a premature termination codon within the LRR domain. This mutation is predicted to result either in nonsense-mediated mRNA decay (NMD) or, if translated, in a severely truncated protein lacking the entirety of the essential regulatory LRR domain. This loss of the LRR regulatory function directly corresponds to one of the established mechanisms (sustained activation due to LRR loss/dysfunction) leading to constitutive *NLRC4* inflammasome activity (7).

This report identifies a novel truncating variant in the *NLRC4* gene (c.1357C > T, p. Arg453*) associated with severe autoinflammatory enterocolitis presenting as NEC in a term neonate. This finding significantly expands the genotypic spectrum of *NLRC4*-associated autoinflammatory diseases (N4AIDs), as the p. Arg453 variant represents the first documented truncating mutation within the LRR domain linked to a severe infantile enterocolitis phenotype. Furthermore, it broadens the associated phenotypic spectrum by demonstrating that a monogenic *NLRC4* inflammasomopathy can manifest as severe intestinal inflammation in a term infant, thereby highlighting a novel genetic etiology for NEC-like presentations in this patient population. This case also had limitations. Due to the retrospective nature of this case report and the unavailability of fresh tissue or viable cells from the patient, we were unable to perform *in vitro* functional validation, and future studies are warranted to validate its functionality. Furthermore, emerging evidence suggests that truncating mutations in genes regulating bacterial immune responses in the neonatal gut may contribute to NEC pathogenesis (21), thereby providing a broader genetic context for our findings regarding *NLRC4*-associated autoinflammatory enterocolitis. Early *NLRC4* screening in neonates may presenting with severe intestinal inflammation, particularly if atypical or familial, facilitate timely diagnosis and guide targeted anti-inflammatory therapies (e.g., IL-1 blockades), potentially improving outcomes.

## Data Availability

The original contributions presented in the study are publicly available. The raw FASTQ files of the genetic sequencing for the family (child, father, mother) have been quality controlled and deposited in the NCBI Sequence Read Archive (SRA) under project accession number PRJNA1468782. This data can be found here: https://dataview.ncbi.nlm.nih.gov/object/PRJNA1468782.
