# Supplementary material for: Autoinflammation with infantile enterocolitis induced by a heterozygous variant (c.1357C > T) in the NLRC4 gene: a case report
Source: Front Pediatr. 2026 May 28;14:1822554. doi: 10.3389/fped.2026.1822554 (PMC13254022; doi:10.3389/fped.2026.1822554)
Supplement: Supplementary file 1 [file Table1.docx]

**Table1: Reported NEC-associated mutation sites and their countries of origin**

| The gene mutation sites that cause NEC | Countries and Regions^[9]^ |
| --- | --- |
| c.620G>A, p.Arg207 Lys | Switzerland |
| c.1010C>A, p.Thr337Asn | Switzerland |
| c.1009A > T, p.Thr337Asn | Europe |
| c.1022T>C, p.Val341Ala | America |
| c.1021G>C, p.Val341Leu | Canada |
| c.1009A > T, p.Thr337Ser | America |
| c.512C> T p.Ser171Phe | America |
| c.1589A>C, p. His443Pro | Japan |
| c.1333T>C p.Ser445Pro | Netherlands |
| c.514G>A p.Gly172Ser | China |
| c.529A>G, p. Thr177Asn | Japan |
| c.1965G>C, p. Trp655Cys | Australia |
| c.1970A> T, p.Gln657Leu | Malaysia |
