# Supplementary material for: Autoinflammation with infantile enterocolitis induced by a heterozygous variant (c.1357C > T) in the NLRC4 gene: a case report
Source: Front Pediatr. 2026 May 28;14:1822554. doi: 10.3389/fped.2026.1822554 (PMC13254022; doi:10.3389/fped.2026.1822554)
Supplement: Supplementary file 2 [file Table2.docx]

**Table2: Candidate genes with preliminary evidence of increased risk for NEC**

| The site of the NEC gene mutation | Functional consequence^[23]^ |
| --- | --- |
| MD2, Promoter SNP (g.73989727C>G) | Unclear |
| SIGIRR, Stop variant (p.Y168X) Missense SNP (p.S80Y; p.P115R) | Loss of function |
| NOD2, Missense SNP (g.19877C>T; g.30491G>C) Insertion/deletion (g.37732_37733insC) | Loss of function |
| MBL, Promoter SNP (−221G>C) | Increased expression |
| NFKB1, Insertion/deletion (g.−24519delATTG) | Decreased expression |
| NFKBIA, Promoter SNP (g.−1004A>G) | Decreased expression |
| IL-6, Promoter SNP (g.4880C>G) | Increased expression |
| VEGF, Missense SNP (C-2578A) | Decreased expression |
| GM2A, Missense SNPs (g.5220G>A; g.19168A>C) | Unclear |
